# Monoclonal antibodies in severe asthma: outcomes from real-world data

**DOI:** 10.3389/fmed.2025.1635688

**Published:** 2025-08-11

**Authors:** Silvia del-Barrio-Buesa, Álvaro Narrillos-Moraza, Julia García-de-Pedro, Francisco Javier de-Castro-Martínez, María Esther Durán-García, Vicente Escudero-Vilaplana, Elena Lobato-Matilla, Rosa Romero-Jimenez, Esther Chamorro-de-Vega, Paula Ruiz-Briones, Félix Jesús Garcia-Moreno, María Martín-Bartolomé, Ana Herranz, María Sanjurjo

**Affiliations:** ^1^Department of Pharmacy, Hospital General Universitario Gregorio Marañón, Madrid, Spain; ^2^Instituto de Investigación Sanitaria Gregorio Marañón, Madrid, Spain; ^3^Department of Pneumology, Hospital General Universitario Gregorio Marañón, Madrid, Spain; ^4^Department of Medicine, Universidad Complutense de Madrid, Madrid, Spain; ^5^Department of Allergy, Hospital General Universitario Gregorio Marañón, Madrid, Spain

**Keywords:** benralizumab, mepolizumab, monoclonal antibody, omalizumab, real-world data, reslizumab, asthma

## Abstract

**Background:**

Uncontrolled severe asthma represents a substantial clinical and economic burden, particularly in patients with comorbidities and poor response to high-dose inhaled corticosteroids. Monoclonal antibodies targeting type 2 (T2) inflammation have become key therapeutic options, but their real-world performance remains insufficiently characterized.

**Objective:**

To evaluate the real-world effectiveness, adherence, and persistence of benralizumab, mepolizumab, omalizumab, and reslizumab in adults with uncontrolled severe asthma after 12 months of treatment.

**Methods:**

A retrospective real-world observational study was conducted in patients with uncontrolled severe asthma who initiated treatment with benralizumab, mepolizumab, omalizumab, and reslizumab between January 2015 and December 2022. Clinical, functional, and laboratory outcomes were assessed at baseline and after 12 months, including eosinophil count, forced expiratory volume in 1 s (FEV_1_), fractional exhaled nitric oxide (FeNO), Asthma Control Test (ACT) score, exacerbations frequency, emergency visits, hospitalizations, adherence, and treatment persistence. Data were extracted from the electronic health record and results are presented as median values with interquartile ranges (IQR).

**Results:**

A total of 154 patients (188 treatment episodes) were included. The median follow-up was 2.2 years (IQR 1.3–4.2). All monoclonal antibodies were associated with significant improvements in asthma control at 12 months. Blood eosinophil counts declined across all therapies, with near-complete depletion observed in patients treated with benralizumab and reslizumab. Median ACT scores increased by six points, and FEV₁ improved by 7%. Annual exacerbation rates and healthcare utilization decreased significantly across all groups. Adherence was high (95%), and the median treatment persistence was 2.0 years (IQR 1.4–4.1). Overall, 42% of patients discontinued treatment, mainly due to insufficient clinical response (48.4%) or drug supply issues (42.2%).

**Conclusion:**

In routine clinical practice, benralizumab, mepolizumab, omalizumab, and reslizumab were associated with improvements in asthma control, lung function, and reduction in exacerbations over 12 months. Benralizumab and reslizumab were associated with the greatest reductions in eosinophil counts. Our findings suggest comparable effectiveness across biologics. High adherence and treatment persistence support their feasibility in real-world settings. These results underscore the relevance of phenotype-driven therapy selection and highlight the need for long-term monitoring to optimize outcomes in severe asthma management.

## 1 Introduction

Asthma is a chronic inflammatory airway disease influenced by genetic and immunological factors. It is characterized by bronchial hyperresponsiveness and variable, partially reversible airflow obstruction. In clinical practice, its management focuses on achieving sustained disease control and preventing exacerbations, which can be life-threatening for the patient and contribute significantly to the healthcare burden (1).

Around 5 to 10% of the total asthmatic population (2–5) is affected by severe asthma (6), a condition associated with increased mortality rates, reduced quality of life, and higher healthcare costs (7). In particular, older adults with asthma constitute a vulnerable population, experiencing more severe symptoms and higher mortality rates. These worse clinical outcomes may be attributed to the presence of chronic comorbidities, which can complicate disease management and progression, reinforcing asthma as a global health concern (8) with several unmet needs in the management of severe asthma (9).

Severe asthma is classified as uncontrolled when, despite optimized treatment with high-dose inhaled corticosteroids plus long-acting *β*₂-agonists, or oral corticosteroids, disease control is not achieved for at least 6 months within a 12-month period, and no other contributing factors aside from disease severity are identified (10). Although a wide range of treatment options is available, this subgroup of patients exhibits an inadequate response to inhaled corticosteroids, often requiring prolonged use of high-dose systemic corticosteroids. This significantly contributes to the economic burden of the disease, estimated at 56 billion US dollars annually, primarily driven by frequent exacerbations requiring acute medical care (11). The costs associated with uncontrolled severe asthma are approximately twice those of controlled severe asthma (4). Consequently, among the broader asthma population, patients with uncontrolled severe asthma represent a group with the greatest unmet medical needs and are potential candidates for monoclonal antibody therapies (12), as recommended by guidelines such as the Global Initiative for Asthma (GINA) (13) and the Spanish Guideline for the Management of Asthma (GEMA) (1).

In patients with uncontrolled severe asthma, identifying the asthma phenotype is a key component of the diagnostic process, as different treatment approaches may be required. The inflammatory patterns have been classified into two main categories: type 2 high (T2- high) and type 2 low (T2-low). Thus, uncontrolled severe asthma can be further categorized into three phenotypes: T2-allergic, T2-eosinophilic, and non-T2. However, some degree of overlap between the two T2 phenotypes is frequently observed (14).

The success of the development of monoclonal antibodies relies on accurately diagnosing the disease phenotype and selecting the appropriate medication (15, 16). Progress in understanding the molecular mechanisms of T2 asthma has resulted in the development of monoclonal antibodies that specifically target immunoglobulins and cytokines involved in the inflammatory cascade (10, 17, 18).

Immunoglobulin E (IgE) was the first therapeutic target for monoclonal antibody treatment in asthma, leading to the development of omalizumab. This agent selectively binds circulating IgE, preventing its interaction with IgE receptors on basophils and mast cells, thereby attenuating the allergic cascade (19). Subsequent monoclonal antibodies have been developed to target other key pathways involved in severe asthma. Interleukin-5 (IL-5), a central mediator of eosinophilic inflammation, is inhibited by mepolizumab and reslizumab. Mepolizumab is a humanized IgG1κ monoclonal antibody that directly targets IL-5 (20), while reslizumab, a humanized IgG4κ monoclonal antibody, blocks IL-5 binding to its receptor (21). Benralizumab is an afucosylated, humanized IgG1κ monoclonal antibody that targets the alpha subunit of the interleukin-5 receptor (IL-5Rα), which is selectively expressed on eosinophils and basophils. The absence of core fucose in its Fc region enhances binding to FcγRIIIa receptors on natural killer cells, thereby augmenting antibody-dependent cell-mediated cytotoxicity (ADCC) and leading to rapid and near-complete depletion of circulating and tissue eosinophils (22).

The latest Global Initiative for Asthma (GINA) 2025 update also recommends dupilumab, which blocks IL-4/IL-13 signaling via IL-4Rα, and tezepelumab, which targets the upstream epithelial cytokine thymic stromal lymphopoietin (TSLP), expanding treatment options for both T2-high and non–T2 asthma phenotypes (13).

Most assessments of these monoclonal antibody therapies come from randomized controlled trials (RCTs), but only a limited number of patients qualify for participation in these studies. RCTs have demonstrated improvements in clinical outcomes with these therapies, but there is a lack of real-world data to support their effectiveness in everyday practice (23, 24) and there are concerns about the applicability of the findings to the general patient population (25, 26).

It has been suggested that the findings from RCTs would gain greater clinical relevance if complemented by evidence of therapeutic effectiveness from routine clinical practice (27). As a result, real-world studies are gaining prominence and increasingly shaping decisions made by regulatory and healthcare authorities (7). Although the pivotal trials for benralizumab (SIROCCO and CALIMA) and mepolizumab (DREAM and MENSA) employed relatively broad eligibility criteria (26, 28), RCTs are conducted in highly controlled settings with standardized management protocols, which may not fully reflect the variability encountered in real-world clinical practice.

There is limited real-world data on monoclonal antibodies to treat uncontrolled severe asthma (24, 26, 27). The objective of this study was to evaluate the use profile and effectiveness of different monoclonal antibodies after 12 months of treatment in patients with uncontrolled severe asthma. Additionally, treatment adherence and persistence were assessed.

## 2 Materials and methods

### 2.1 Study design

A longitudinal, retrospective, observational study was conducted. The study included patients over 18 years old diagnosed with uncontrolled severe asthma who started treatment with benralizumab, mepolizumab, omalizumab, or reslizumab under clinical practice conditions from January 2015 to December 2022 at a tertiary hospital in Spain. Patients were followed from treatment initiation until March 2024. Those with a treatment duration of less than 1 year were excluded from the study.

Monoclonal antibodies included were benralizumab, mepolizumab, omalizumab, and reslizumab, all of which are classified under ATC Code R03DX: Other systemic drugs for obstructive airway diseases.

The study was carried out according to the Declaration of Helsinki and was approved by the Research Ethics Committee.

### 2.2 Variables and data recorded

Variables recorded at treatment initiation:

- Demographic variables: Age, sex, body mass index (BMI).
- Clinical variables: Types of comorbidities.
- Monoclonal antibody therapy: Type and start date of treatment.
- Laboratory parameter: Eosinophil level (cells/μL).
- Respiratory function parameters: Forced expiratory volume in the first second (FEV_1_ (%)) and fractional exhaled nitric oxide (FeNO) levels.
- Asthma control parameters: Asthma Control Test (ACT) score and the number of systemic glucocorticoid cycles per year due to asthma exacerbations.
- Healthcare utilization parameters: Number of hospital admissions and emergency visits in the past 12 months.

Variables recorded during the first 12 months of treatment:

- Laboratory parameter: Eosinophil level (cells/μL).
- Respiratory function parameters: FEV_1_ (%) and FeNO levels.
- Asthma control parameters: ACT score and the number of systemic glucocorticoid cycles due to asthma exacerbations. Asthma exacerbations were defined as those requiring oral corticosteroid doses or an increase in oral corticosteroid dosage for ≥3 days.
- Healthcare utilization parameters: Number of hospital admissions and emergency visits.

Treatment adherence was defined as the medication possession ratio from the beginning of treatment to the end of the follow-up period. This evaluation was conducted using the hospital’s patient registry, which includes outpatients who self-administer the medication at home and patients receiving intravenous medication at the day hospital. Treatment persistence was defined as the time from treatment initiation to discontinuation of the monoclonal antibody. The reasons for discontinuation were also recorded.

Effectiveness was assessed based on the outcomes of the recorded variables after the first 12 months of treatment: eosinophil levels, percentage of forced expiratory volume in 1 s (FEV_1_ (%)), asthma control test (ACT) score, and number of hospitalizations and emergency visits. Differences in clinical data, pulmonary function, and laboratory parameters were assessed across the four monoclonal antibodies at baseline and after 12 months of treatment. When significant differences between monoclonal antibody types were identified, additional statistical analyses were performed to determine which specific treatments were associated with these outcomes.

### 2.3 Statistical analysis

The statistical analysis was performed based on treatment episodes, defined as the continuous period during which a patient received the same monoclonal antibody without discontinuation. Four types of monoclonal antibodies were analysed: benralizumab, mepolizumab, omalizumab, or reslizumab.

A descriptive analysis of the sample was conducted, and normality was assessed using the Shapiro–Wilk test (for *n* < 50) and the Shapiro-Francia test (for *n* > 50). Additionally, a comparative analysis of both numerical and categorical variables was performed. Medians and interquartile ranges (IQR) were used as measures for continuous variables, while categorical variables were presented as counts (frequency) and percentages.

Fisher’s exact test was used to compare categorical variables. For the overall comparison of numerical variables across the four monoclonal antibody treatments, the Kruskal–Wallis test was applied (a *p*-value ≤0.05 indicates significant differences between monoclonal antibodies).

Finally, for variables with statistically significant differences (*p* ≤ 0.05), multiple comparisons among the four monoclonal antibody groups were conducted using the Mann–Whitney U test. All six possible pairwise comparisons were evaluated. Median values and their 95% confidence intervals (CIs) were estimated using non-parametric bootstrap resampling (10,000 replicates with replacement; 2.5th–97.5th percentiles; seed = 42).

## 3 Results

### 3.1 Patient demographic and pharmacotherapeutic profile

A total of 154 patients diagnosed with uncontrolled severe asthma were included in the study, accounting for 188 different treatment episodes, as some patients required a switch to another monoclonal antibody. The monoclonal antibodies were benralizumab (*n* = 20), mepolizumab (*n* = 96), omalizumab (*n* = 34), and reslizumab (*n* = 38). The median duration of patient follow-up was 2.2 years (IQR 1.3–4.2).

The median age of the patient cohort was 55 years (IQR 46–67) with 77.7% being women, and the median body mass index was 28.1 kg/m^2^ (IQR 24.4–32.5). The comorbidities identified included obesity, nasal polyposis, rhinosinusitis, atopy, chronic obstructive pulmonary disease (COPD), gastroesophageal reflux disease, and a history of smoking. The rest of baseline patient demographic, comorbidities, and clinical characteristics are detailed in Table 1.

**Table 1:** Patient baseline profile.

Characteristics	All monoclonal antibody therapies *n* = 188	Omalizumab *n* = 34	Mepolizumab *n* = 96	Reslizumab *n* = 38	Benralizumab *n* = 20	Comparison of the 4 treatment groups, *p-*value
Age, years, median (IQR)	55 (46–67)	45.5 (38–57)	59 (50–69)	51.5 (42–67)	62.5 (52.5–75)	0.000
Women, *n* (%)	146 (77.7)	25 (73.5)	68 (70.8)	35 (92.1)	14 (70.0)	0.067
BMI, kg/m^2^, median (IQR)	28.1 (24.4–32.5)	28.3 (24.0–31.2)	29.0 (24.0–31.2)	23.9 (21.8–28)	30.7 (27.9–37.5)	0.000
Comorbidities, *n* (%)						
Obesity	71 (37.8)	9 (13.0)	43 (44.8)	6 (15.8)	11 (55.0)	0.004
Ex-smokers	62 (33.0)	11 (32.3)	35 (36.5)	9 (23.7)	7 (35.0)	0.563
Nasal polyposis	71 (37.8)	9 (26.5)	36 (37.9)	19 (50.0)	7 (35.0)	0.231
Rhinosinusitis	116 (61.7)	24 (70.6)	58 (61.0)	23 (60.5)	9 (45.0)	0.327
Atopy	99 (52.7)	28 (82.3)	40 (41.7)	17 (44.7)	10 (50.0)	0.000
COPD	10 (5.3)	0 (0.0)	7 (7.4)	2 (5.3)	1 (5.0)	0.444
Gastroesophageal reflux	56 (29.8)	11 (32.3)	32 (33.7)	8 (21.0)	4 (20.0)	0.378
Laboratory parameters						
Historical maximum of eosinophils (cells/μL), median (IQR)	800 (500–1,300)	900 (500–1,300)	800 (500–1,300)	800 (500–1,400)	650 (400–900)	0.349
Respiratory functional parameters						
FEV 1, %, median (IQR)	77 (63–88.2)	74.5 (67–93.1)	76 (60.5–87.5)	76 (66–88)	77 (56–90)	0.829
FeNO, ppb, median (IQR)	40 (23–62)	44.2 (27.5–67)	29.7 (17.5–61)	39.5 (24–77)	43.4 (24–76)	0.431
Asthma control parameters						
ACT, median (IQR)	15 (11–18)	12 (11.5–15.5)	15 (10–18)	16 (11–18)	13.5 (11–17)	0.450
Exacerbations/year, median (IQR)	2 (1–3)	2.5 (1–3.5)	2 (1–3)	2 (1–4)	1.5 (0–2.5)	0.034
Previous hospital admissions/year, median (IQR)	1 (0–2)	2 (1–3)	1 (0–1)	2 (1–2)	1 (0.5–2)	0.000

*n*, number of patients; BMI, body mass index; IQR, interquartile range; COPD, chronic obstructive pulmonary disease; FEV_1,_ forced expiratory volume in the first second; FeNO, fractional exhaled nitric oxide; ACT, asthma control test.

Eosinophil levels were highest in patients who started treatment with omalizumab: 900 cells/μL (IQR 500–1,300), whereas those who started treatment with benralizumab had the lowest eosinophil levels: 650 cells/μL (IQR 400–900). However, no statistically significant differences were found among the four monoclonal antibodies (*p* = 0.349) (Table 1).

We found that omalizumab was more commonly used in patients with atopy (*n* = 28, 29.5%; *p* < 0.001), whereas mepolizumab was more frequently used in patients without atopy (*n* = 56, 60.2%; *p* = 0.0139). We observed that reslizumab was more frequently used in non-obese patients (*n* = 32, 27.8%; *p* = 0.0023) and in women (*n* = 35, 92.1%; *p* = 0.0061).

Statistically significant differences were found in baseline annual exacerbations (*p* = 0.034) and baseline hospital admissions and emergency visits in the last year before starting monoclonal antibody treatment (*p* < 0.001). Asthma control was poor with a median ACT score of 15 (IQR 11–18).

### 3.2 Outcomes after 12 months of treatment

**Figure 1:**
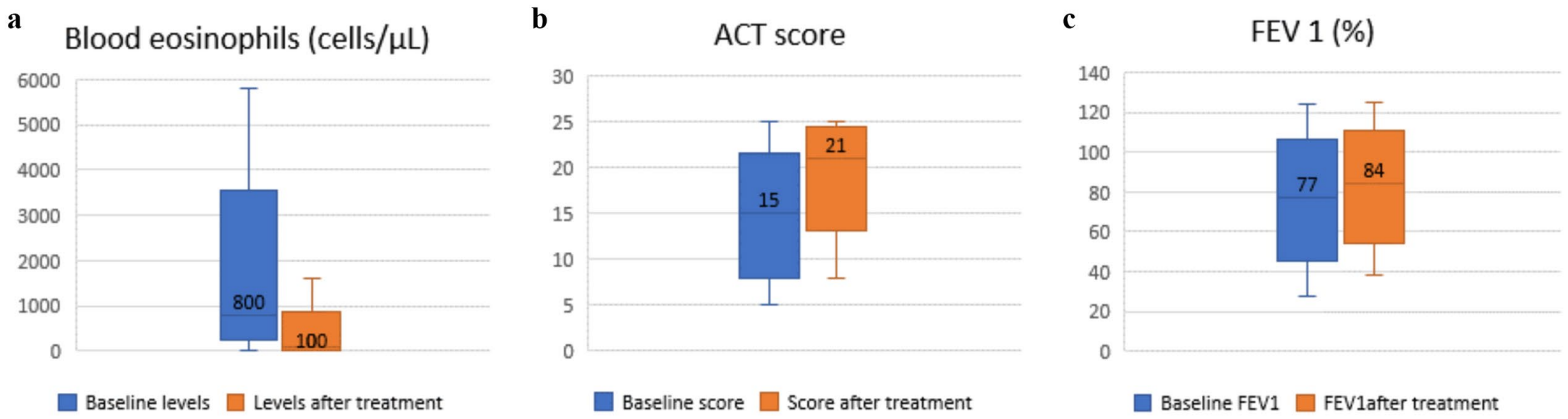
Changes in eosinophil count, ACT score and FEV_1_ from baseline to 12 months after monoclonal antibody therapy. **(a)** Changes in eosinophil count from baseline to 12 months after monoclonal antibody therapy. **(b)** Changes in ACT score from baseline to 12 months after monoclonal antibody therapy. **(c)** Changes in FEV_1_ from baseline to 12 months after monoclonal antibody therapy.

After 12 months of treatment, a reduction in total eosinophil levels (−700 cells/μL) was observed (Figure 1a), and the median reduction in blood eosinophils was consistent across all monoclonal antibodies: −650 cells/μL (95% CI − 750 to −450) with benralizumab, −700 cells/μL (95% CI − 800 to −500) with mepolizumab and omalizumab (95% CI − 900 to −400), and −800 (95% CI − 1,100 to −600) cells/μL with reslizumab.

Eosinophil depletion was complete in patients treated with benralizumab, while in those receiving reslizumab, the median eosinophil count was 0 cells/μL (IQR 0–100). In contrast, eosinophil counts decreased to a median of 200 cells/μL (IQR 100–500) with omalizumab, and to 100 cells/μL (IQR 0–100) with mepolizumab, *p* < 0.001.

An overall increase in Asthma Control Test (ACT) scores (+6 points) (Figure 1b) was found. ACT score increases by +7.5 with benralizumab, +6 with mepolizumab, +9.5 with omalizumab, and +6 with reslizumab. However, no significant differences were observed among the four types of monoclonal antibodies.

An improvement in lung function was also observed, reflected by an increase in FEV_1_ (+7%) (Figure 1c). All monoclonal antibody treatments showed improvements in FEV_1_, +1% with benralizumab, +10% with mepolizumab, +16.5% with omalizumab, and +3% with reslizumab. However, no statistically significant differences were observed among the four types of monoclonal antibodies.

Additionally, a reduction in the annual number of exacerbations was observed across all therapies, from two per year (IQR 1–3) to none (IQR 0–1). The annual reduction in exacerbations was −1.5 with benralizumab, −2 with mepolizumab, −2.5 with omalizumab, and −2 with reslizumab (Table 2). Similarly, a decrease in emergency visits and asthma-related hospitalizations was observed for all therapies, from one per year (IQR 0–2) to none (IQR 0–1). However, no statistically significant differences were found (*p* = 0.845 and *p* = 0.352, respectively).

**Table 2:** Outcomes after 12 months of monoclonal antibody therapy.

Characteristics	All monoclonal antibody therapies *n* = 188	Omalizumab *n* = 34	Mepolizumab *n* = 96	Reslizumab n = 38	Benralizumab *n* = 20	Comparison of the 4 treatment groups, *p-*value
Laboratory parameters						
Level of eosinophils (cells/μL) median (IQR)	100 (0–100)	200 (100–500)	100 (0–100)	0 (0–100)	0 (0–0)	0.000
Respiratory functional parameters						
FEV 1, %, median (IQR)	84 (71–97)	91 (77.6–103)	86 (73–97)	79 (66–91)	78 (60–94)	0.071
FeNO ppb, median (IQR)	33.1 (23.8–51.7)	32.6 (11.7–79.4)	32 (18.7–69.5)	33.25 (26.6–50.4)	38 (32–49)	0.964
Asthma control parameters						
ACT, median (IQR)	21 (18–24)	21.5 (19–24)	21 (19–24)	22 (19–23)	21 (16.5–24.5)	0.969
Exacerbations/year, median (IQR)	0 (0–1)	0 (0–1)	0 (0–1)	0 (0–0)	0 (0–1)	0.845
Hospital admissions/year, median (IQR)	0 (0–1)	0 (0–0)	0 (0–0)	0 (0–0)	0 (0–1)	0.352

*n*, number of patients; IQR, interquartile range; FEV_1_, forced expiratory volume in the first second; FeNO, fractional exhaled nitric oxide; ACT, asthma control test.

Treatment adherence was 95%. The median treatment persistence was 2.0 years (IQR 1.4–4.1), with median treatment durations of 0.4 years for benralizumab, 2 years for mepolizumab, 4.1 years for omalizumab and 1.8 years for reslizumab.

A total of 64 patients (42%) discontinued the monoclonal antibody treatment during the follow-up period: 30 patients (47%) reslizumab, 20 (31%) omalizumab, 11 (17%) mepolizumab, and 3 (5%) benralizumab. Among patients who discontinued treatment, the most common reason was insufficient clinical efficacy (31 patients, 48.4%) associated with worsening lung function. Additionally, two patients (3.1%) discontinued treatment due to safety concerns, such as skin reactions, hypotension, dizziness, fever, and cough; four patients discontinued treatment due to work-related reasons or loss of follow-up (6.3%); and 30 patients (42.2%) due to reslizumab shortage.

### 3.3 Multiple comparisons among different monoclonal antibodies

Nominally significant differences were observed in the following variables: baseline exacerbations per year, baseline hospital admissions per year, and eosinophil levels after 12 months of monoclonal antibody treatment (Table 3).

**Table 3:** Multiple comparisons between the four types of monoclonal antibodies.

Characteristics	*p*-value Omalizumab *vs* Mepolizumab	*p-*value Omalizumab *vs* Reslizumab	*p*-value Omalizumab *vs* Benralizumab	*p-*value Mepolizumab *vs* Reslizumab	*p-*value Mepolizumab *vs* Benralizumab	*p*-value Reslizumab *vs* Benralizumab
Baseline exacerbations/year	0.011	0.251	0.019	0.185	0.493	0.138
Baseline hospital admissions/year	0.000	0.357	0.188	0.002	0.076	0.467
Level of eosinophils after 12 months	0.000	0.000	0.000	0.031	0.006	0.210

These comparisons were only performed for variables with a *p*-value ≤0.05. All possible pairwise comparisons (*n* = 6) were performed using the Mann–Whitney U test.

For baseline annual exacerbations, nominally significant differences were found between:

- Mepolizumab vs. omalizumab: 2 (IQR 1–3) vs. 2.5 (IQR 1–3.5), p = 0.011.
- Benralizumab vs. omalizumab: 1.5 (IQR 0–2.5) vs. 2.5 (IQR 1–3.5), p = 0.019.

For baseline hospital admissions and emergency visits, nominally significant differences were found between:

- Mepolizumab vs. omalizumab: 1 (IQR 0–1) vs. 2 (IQR 1–3), p < 0.001.
- Mepolizumab vs. reslizumab: 1 (IQR 0–1) vs. 2 (IQR 1–2), p = 0.002.

For eosinophil levels after 12 months of treatment, nominally significant differences among all the types of monoclonal antibodies were found, except between reslizumab and benralizumab (*p* = 0.210).

Given the number of pairwise tests and the limited sample size in some groups, these results should be interpreted as exploratory and hypothesis-generating rather than confirmatory.

## 4 Discussion

This study shows real-world effectiveness of monoclonal antibodies in the treatment of uncontrolled severe asthma in a cohort of 154 patients (188 treatment episodes) over a 12-month period. All treatment groups appeared to show clinical improvement over the 12-month period, as suggested by trends toward better asthma control and reduced healthcare utilization. High adherence and moderate treatment persistence further support the feasibility of these therapies in routine clinical practice.

A key distinction from RCTs is that our cohort includes patients with multiple comorbidities, a group consistently under-represented in RCTs (28–30). Even though the pivotal benralizumab trials (SIROCCO, CALIMA) and mepolizumab trials (DREAM, MENSA) broadened their eligibility criteria, the enrolled populations still capture only a small fraction of patients treated in routine practice. Published analyses estimate that RCT participants represent merely 5–10% of real-world patients with severe asthma (26). In one observational cohort of 119 adults receiving mepolizumab, 82% would have been excluded from those trials because of advanced age, current smoking, severe obesity, or respiratory comorbidities such as COPD or bronchiectasis (28). These figures highlight the persistent gap in external validity and reinforce the rationale for the present real-world study.

At baseline characteristics, the various monoclonal antibody therapies showed statistically significant differences in patient age and body mass index, as well as in comorbidities such as atopy and obesity, the latter being a condition that requires intensive management in patients with asthma (25). Mepolizumab was the most frequently used monoclonal antibody in our study, in line with previous real-world evidence (30), and was significantly more prescribed in patients with obesity. Reslizumab was more frequently prescribed in non-obese patients and women, possibly due to its weight-based dosing regimen. Moreover, we observed that omalizumab was more commonly prescribed in patients with atopy. It may be explained by the presence of elevated IgE levels, which are closely associated with other allergic diseases. Although only a small proportion of patients in our cohort had a diagnosis of COPD (5.3%) or a history of smoking (33.0%), these factors are known to influence treatment response in severe asthma (28). In our study, the low prevalence of COPD and its balanced distribution across treatment groups likely minimized any potential confounding impact on the overall outcomes.

The greater FEV₁ gain observed with omalizumab may be attributable to a younger, less-obese, highly atopic patient profile combined with the longest treatment exposure (median 4.1 years). Despite this, no significant differences were found among the four monoclonal antibodies regarding eosinophil levels, FEV_1_ and FeNO values, and ACT scores. According to these data, all patients seemed to be equally symptomatic at baseline. However, significant differences were found in the number of annual exacerbations and the number of hospital admissions and emergency visits. Patients receiving omalizumab and reslizumab exhibited a higher number of annual exacerbations, hospital admissions, and emergency visits, which may suggest a more severe disease in these patients and evidence poorer disease control.

At 12 months after the start of treatment, all monoclonal antibodies showed improvement in critical related asthma outcomes, such as enhanced lung function, improved asthma control, reduced frequency of exacerbations, and hospitalizations and emergency visits. These findings are consistent with previous studies (31, 32), and support the use of these treatments in patients with severe uncontrolled asthma, particularly when the goal is to reduce the exacerbation rate or the use of oral corticosteroids (33). In consequence, the management of patients with asthma-associated comorbidities using a monoclonal antibody that can also control these conditions could be a cost-saving approach (34). Other real-world studies on mepolizumab and benralizumab have also shown significant reductions in annual exacerbation rates after treatment initiation, leading to both clinical improvements and economic benefits by lowering asthma-related healthcare costs (35–37), associated with significant reductions in hospital admissions and emergency visits.

Additionally, significant differences were found in the reduction of eosinophil levels after 12 months of treatment, except between benralizumab and reslizumab where lower eosinophil counts were found. Patients receiving benralizumab showed the lowest blood eosinophil counts, in line with findings from previous real-world studies (28, 31, 38). Although significant differences were observed in blood eosinophil depletion among treatments, all four drugs contributed to a reduction in asthma exacerbations, a decrease in emergency visits and asthma-related hospitalizations, and an improvement in lung function, as evidenced by an increase in FEV_1_ values. However, consistent with previous studies, no reductions in FeNO were observed across the four monoclonal antibodies despite improvements in clinical symptoms and FEV_1_ values. FeNO values should be interpreted alongside the patient’s clinical status and other pulmonary function tests (7, 39). The lack of effect of these treatments on FeNO levels can be explained by the fact that nitric oxide is primarily stimulated by pro-inflammatory T2 cytokines such as IL-4 and IL-13 (40). Its role as a biomarker for clinical control is not yet well established and remains an area for future research (41).

Poor treatment adherence is a well-known risk factor for asthma exacerbations, increased mortality, and higher healthcare resource utilization (42, 43). However, adherence in the study was high (95%), supporting its effectiveness. The potentially high treatment adherence highlights the significance of the observed reductions in exacerbation rates, consistent with findings from previous studies (44).

Treatment persistence could be considered as an indirect indicator of both effectiveness and safety (45). The estimated median persistence in our study was 2.0 years (IQR 1.4–4.1). Compared to a prior study (45), these findings indicate that patients maintain the treatment long-term, possibly due to its clinical benefits and positive health outcomes. However, there is limited evidence to properly interpret this persistence period accurately (45, 46) as it is influenced by factors related to the patient, the provider, and the healthcare system. Additionally, the shortage of reslizumab may affect the representativeness of these data.

It is noteworthy that 42% of patients in our study discontinued treatment. Despite the high rate of discontinuation, these results are consistent with other real-world studies. Maddux et al. evaluated treatment persistence among 9,575 patients receiving biologics for asthma and found that only 45% remained on therapy for at least 12 months (45). In our cohort, the most common reasons for discontinuation were suboptimal clinical efficacy and adverse events, in line with previous studies (31, 47, 48). Additionally, a temporary shortage of reslizumab contributed to treatment interruptions, reflecting logistical challenges often encountered in routine clinical practice rather than issues related to therapeutic performance. Importantly, only 3.1% of patients discontinued due to safety concerns, which included skin reactions, hypotension, dizziness, fever, and cough. Among the therapies analyzed, mepolizumab and benralizumab were associated with the lowest discontinuation rates.

The main limitation of this study is its single-center design, which restricts the generalizability of the findings to other clinical settings. Nonetheless, our primary objective was to provide insight into real-world clinical practice by examining how these treatments are used across diverse patient profiles, evaluating their effectiveness, and analyzing treatment adherence, persistence, and reasons for discontinuation. Another limitation is the exclusion of dupilumab from the analysis. Although dupilumab is an important therapeutic option for patients with T2-high asthma, it was not reimbursed for asthma in the Spanish national formulary until February 2022. As a result, no patients in our cohort had completed at least 12 months of treatment with dupilumab during the inclusion period.

Additional limitations include the regulatory restrictions associated with each biologic’s approved indication (e.g., high total IgE for omalizumab, elevated blood eosinophils for mepolizumab and benralizumab, or FeNO ≥ 25 ppb for dupilumab), which may bias the study population toward atopic or eosinophilic phenotypes. The relatively small sample size further limits the statistical power to detect subtle differences between treatment subgroups. Finally, the long study period (2015–2022) spans a time of evolving clinical practice, including the expanded use of inhaled LAMAs and the introduction of single-inhaler triple therapy. These changes may represent unmeasured confounding factors that could influence treatment outcomes.

## 5 Conclusion

This real-world study provides supportive evidence on the clinical utility of monoclonal antibodies in reducing eosinophilic inflammation and improving asthma control in patients with uncontrolled severe asthma, including those with complex comorbidities often underrepresented in randomized controlled trials. Observed reductions in blood eosinophil counts, improvements in FEV₁ and ACT scores, and decreased healthcare utilization suggest a potential therapeutic benefit in routine clinical settings.

Benralizumab and reslizumab were associated with the greatest reductions in eosinophil counts, while mepolizumab showed consistent improvements in patients with obesity. Although our findings suggest that all biologics provided comparable clinical benefits after 12 months of treatment, these results should be interpreted cautiously given the limited sample size and exploratory nature of the comparisons.

High adherence and moderate treatment persistence further support the feasibility of biologic therapies in real-world practice. Nonetheless, treatment discontinuation, particularly linked to limited reslizumab availability, highlights the importance of ensuring continuous access and close monitoring during long-term therapy.

Overall, our findings support a phenotype-driven, personalized approach to biologic therapy in severe asthma and reinforce the relevance of real-world data in guiding clinical decisions. Future multicenter studies with larger sample sizes and longer follow-up are needed to confirm these trends and better define the long-term effectiveness and safety of these treatments.

## Data Availability

The original contributions presented in the study are included in the article/supplementary material, further inquiries can be directed to the corresponding author.
